# The diabetic microenvironment causes mitochondrial oxidative stress in glomerular endothelial cells and pathological crosstalk with podocytes

**DOI:** 10.1186/s12964-020-00605-x

**Published:** 2020-07-08

**Authors:** Gabriella A. Casalena, Liping Yu, Roberto Gil, Samuel Rodriguez, Shantel Sosa, William Janssen, Evren U. Azeloglu, Jeremy S. Leventhal, Ilse S. Daehn

**Affiliations:** 1 Division of Nephrology, Department of Medicine, The Icahn School of Medicine at Mount Sinai, One Gustave Levy Place, Box 1003, New York, NY 10029 USA; 2 Microscopy CoRE, The Icahn School of Medicine at Mount Sinai, New York, USA

**Keywords:** Endothelial cells, Diabetes, Crosstalk, Podocytes, Mitochondria, ROS

## Abstract

**Background:**

In the setting of diabetes mellitus, mitochondrial dysfunction and oxidative stress are important pathogenic mechanisms causing end organ damage, including diabetic kidney disease (DKD), but mechanistic understanding at a cellular level remains obscure. In mouse models of DKD, glomerular endothelial cell (GEC) dysfunction precedes albuminuria and contributes to neighboring podocyte dysfunction, implicating GECs in breakdown of the glomerular filtration barrier. In the following studies we wished to explore the cellular mechanisms by which GECs become dysfunctional in the diabetic milieu, and the impact to neighboring podocytes.

**Methods:**

Mouse GECs were exposed to high glucose media (HG) or 2.5% v/v serum from diabetic mice or serum from non-diabetic controls, and evaluated for mitochondrial function (oxygen consumption), structure (electron microscopy), morphology (mitotracker), mitochondrial superoxide (mitoSOX), as well as accumulation of oxidized products (DNA lesion frequency (8-oxoG, endo-G), double strand breaks (γ-H2AX), endothelial function (NOS activity), autophagy (LC3) and apoptotic cell death (Annexin/PI; caspase 3). Supernatant transfer experiments from GECs to podocytes were performed to establish the effects on podocyte survival and transwell experiments were performed to determine the effects in co-culture.

**Results:**

Diabetic serum specifically causes mitochondrial dysfunction and mitochondrial superoxide release in GECs. There is a rapid oxidation of mitochondrial DNA and loss of mitochondrial biogenesis without cell death. Many of these effects are blocked by mitoTEMPO a selective mitochondrial anti-oxidant. Secreted factors from dysfunctional GECs were sufficient to cause podocyte apoptosis in supernatant transfer experiments, or in co-culture but this did not occur when GECs had been previously treated with mitoTEMPO.

**Conclusion:**

Dissecting the impact of the diabetic environment on individual cell-types from the kidney glomerulus indicates that GECs become dysfunctional and pathological to neighboring podocytes by increased levels of mitochondrial superoxide in GEC. These studies indicate that GEC-signaling to podocytes contributes to the loss of the glomerular filtration barrier in DKD.

Video abstract

**Graphical abstract:**

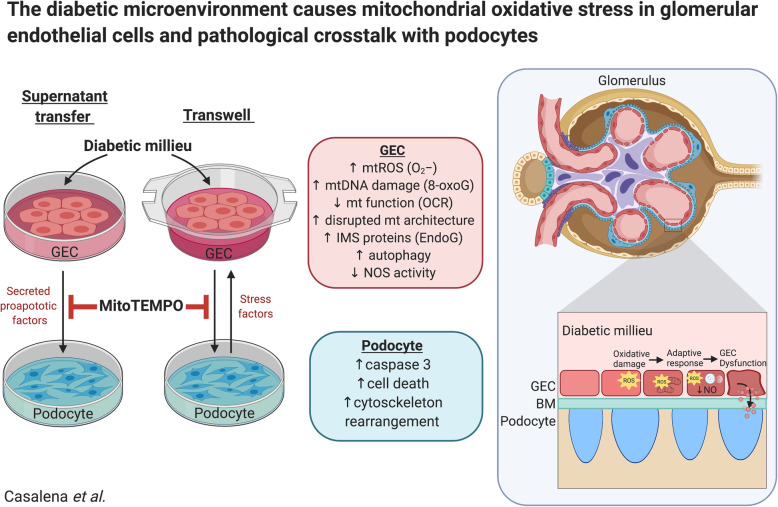

## Plain English summary

Diabetic kidney disease is one of the major causes of death in diabetic patients and also the leading single cause of end stage renal disease in the United States. It is a major public health problem, both in terms of disease burden and health expenditure as it can lead to kidney failure and eventual need for dialysis or kidney transplantation. Unfortunately the prevalence of diabetic kidney disease continues to grow to epidemic proportions worldwide, so efforts to stop or reverse disease progression have the potential to significantly impact many patients suffering from this debilitating disease.

The communication between cells in the glomerulus (filtration unit of the kidney) in response to injury from the diabetic circulation is critical to trigger disease progression, however there is very little known about such intercellular communication. In this study we show that glomerular endothelial cells become stressed and dysfunctional in a diabetic environment, and that prolonged exposure results in the release of soluble injury signals that affect podocyte health. Blocking endothelial cell stress, prevented the damaging signals to podocytes. The studies presented pinpoint key events in each cell type and provide important clues for the exploration of more effective therapies for DKD.

## Background

The glomerulus is early and important site of diabetic injury in the kidney. Glomerular hypertrophy, basement membrane thickening, capillary loop obliteration and loss of podocytes from the glomerular filtration barrier are hallmarks of progressive DKD, while the degree of podocyte loss correlates with severity of the disease [1–4]. In diabetes, glomerular endothelial cell (GEC) injury is an early event, detectable before the onset of albuminuria, and has been proposed to contribute to DKD by release of paracrine mediators affecting mesangial cells and podocytes leading to subsequent glomerular injury [5–7]. The molecular mechanisms for glomerular cell crosstalk and feedback regulation in proteinuric glomerular diseases remain poorly understood.

Reactive oxygen species (ROS) are a potential direct mechanism in the pathogenesis in diabetes and its complications including DKD [2, 8]. Factors including an increase in mitochondria ROS (mtROS mainly superoxide), mitochondrial dysfunction with decreased ATP production, perturbed balance of mitochondrial dynamics, decreased mitochondrial turnover, enhanced fatty acid oxidation and altered glycolytic flux play a critical role in DKD [9–12]. We have demonstrated that mitochondrial superoxide and oxidative stress damage accumulates particularly in GEC in diabetic mice that are susceptible to developing DKD (DKD susceptible DBA2/J mice), but not in diabetic mice that do not develop DKD (DKD resistant C57BL/6 mice) [13]. We have also shown that deleterious endothelial-to-podocyte crosstalk occurring in the setting of cell stress may underlie segmental lesions in experimental models of FSGS [14, 15]. The context of mitochondrial stress is important as previous studies in which endothelial cells and podocytes were purified from glomeruli of mice after induction of diabetes, confirmed mitochondrial dysfunction and oxidative stress pathways were enriched selectively in GECs whereas podocytes demonstrated distinct changes in the regulation of actin cytoskeleton-related genes [16, 17].

Mitochondrial oxidative stress in GECs may result from the combined exposure to noxious stimuli including high glucose, uric acid, fatty acids, growth factors, cytokines, angiotensin, endothelin-1 and hormones in the diabetic milieu, and contribute to the glomerular injury observed in DKD. Excessive production of mitochondrial ROS can damage macromolecules within mitochondria, including lipids, proteins as well as mtDNA [18–21]. This in turn impairs the synthesis of components of the electron transport chain, and reduces the capacity to generate energy in form of ATP, further potentiating ROS production (vicious cycle: oxidative stress) and resulting in mitochondrial dysfunction [19, 22]. An increased production of mitochondrial superoxide was depicted in real-time in the kidneys of diabetic mice [23]. Mitochondrial stress in DKD was shown to be linked to induction of Rho-associated coiled-coil-containing protein kinase1 (ROCK1) expression, which resulted in mitochondrial fission and mitochondrial dysfunction in both podocytes and glomerular endothelial cells [24]. Mitochondrial quality control mechanisms are crucial for efficient mitochondrial function in health and also play a fundamental role in disease [25, 26]. Autophagy is a cellular process that is responsible for removing protein aggregates and damaged or excess organelles to maintain intracellular homeostasis and cell integrity. In an effort to preserve energy production, damaged mitochondria undergo a specialized process of cellular autophagy known as mitophagy [27]. When damaged mitochondria lose membrane potential, they are sequentially labelled with PTEN-induced putative kinase protein 1 and then with an E3 ubiquitin-protein ligase (Parkin) thus facilitating the formation of an autophagosome around the damaged mitochondria [28]. There is evidence of accumulation of autophagosomes containing mitochondria in human diabetic kidneys and in rodent models of DKD [29, 30]. Reports also indicate that the autophagic machinery may be disrupted in DKD [31, 32], suggesting that the mitochondrial turnover pathways could impact energy production and consequently reduce renal function in DKD.

Here we evaluated the impact of the diabetic microenvironment on GECs. Prolonged exposure to diabetic serum recapitulates deleterious effects on mitochondrial health resulting in cellular dysfunction, but not cell death. We hypothesize that cells may enter an adaptive state of cell dysfunction, which promotes the secretion of proapoptotic factors and pathogenic crosstalk with podocytes.

## Methods

### Cell culture

The conditionally immortalized mouse podocytes or murine glomerular endothelial cells (mGECs) were maintained in RPMI-1640 with L-glutamine (Invitrogen) containing 10% FBS and 1% penicillin/streptomycin on collagen I coated flasks under either permissive (33 °C with 10 units/ml of INF-γ), or non-permissive condition (37 °C without INF-γ) in a humidified incubator (95% air, 5% CO2). mGECs were incubated with normal glucose (5 mM) + D-mannitol (25 mM), or with high glucose (HG; 30 mM D-Glucose), or with control or diabetic serum (2.5%v/v), without or with MitoTEMPO (100 nM, Enzo Life Sciences International, PA) for up to 72hs. Transwell co-cultures were performed in collagen-I coated 6x well plates and 0.4um 24 mm inserts (Costar).

### Mice sera

Eight weeks old inbred DBA/2 J and C57BL/6 male mice (Jackson Laboratory) received low-dose streptozotocin (STZ) (50 mg/kg) injections intraperitoneally for five consecutive days [13, 33]. STZ was freshly made in 0.1 mol/L sodium citrate buffer, PH4.5. The onset and extent diabetes were evaluated by weekly measurement of 4–6 h fasting blood glucose (FBG) using blood glucose strips and meter (Ascensia Contour, Bayer healthcare LLC). Hyperglycemia was considered when FBG > 400 mg/dl. Blood serum was collected after 6 week of established hyperglycemia as well as from untreated control mice. All animal protocols and procedures were approved by IACUC at The Icahn School of Medicine at Mount Sinai.

### Plasmids and transfections

Wild-type GFP-endonuclease-G cloned in pEGFP-N1 plasmid vector was kindly provided by Dr. Doris Germain. Constructs were transformed in plasmid vector DH5α *E. coli* cells and plated on kanamycin plates for selection overnight at 37 °C. Selected colonies were placed in 3 ml of LB broth + kanamycin overnight at 37 °C with shake (240 rpm). We used Illustra Plasmidprep Mini Spin Kit to extract and purify the plasmid DNA from the small scale cultures of E.coli, and then DNA was quantified using a Nanodrop One. All transfections were conducted using the Lipofectamine 3000 Reagent, as per manufacturer (ThermoFisher Scientific).

### Western blotting

Cultured mGEC or podocyte lysates were made using RIPA buffer containing protease inhibitor cocktail (Roche) and Halt phosphatase inhibitor (Thermo Scientific). Protein quantification by Pierce BCA protein assay (ThermoFisher). After SDS-PAGE gel fractionation and membrane transfer, the blots were blocked and incubated with primary antibodies against anti-GFP (Abcam), LC3 (MBL International), cleaved caspase 3 (Santa Cruz) and β-actin (Santa Cruz Biotechnology) and secondary antibodies conjugated w/ HRP (Cell Signaling, Beverly, MA). Immunoblots were developed by Enhanced Chemiluminescence (ECL). Bands were analyzed using ImageJ software (NIH).

### Immunofluorescence staining and microscopy

Mitotracker® Red CMXRos (Thermo Fisher Scientific) to visualize mitochondrial networks in live mGECs [34].

mGECs on coverslips were fixed with methanol and incubated with anti-8-oxoG monoclonal antibody (N45.1; Japan Institute for the Control of Aging) previously described [35]. Cells were also stained with either goat anti-mtTFA antibodies (Santa Cruz), rabbit anti-LC3 (Novus Biologicals), or rabbit anti-phospho-histone H2A.X (Ser139) Antibody (Cell Signaling Technology). The antigen-antibody complexes were visualized with Alexa Fluor secondary antibodies. DAPI was added to mounting medium. mGECs plated in 12-well slide (Ibidi) at a density of 2.0 × 10^4^ cells/well and were transfected with WT and mutant GFP-endoG and fixed with 4% PFA in PBS. DAPI was added to mounting medium. Podocytes on coverslips were fixed with 4% PFA in PBS, incubated anti-Caspase 3 antibodies (cleaved Santa Cruz) and Phalloidin-FITC (Sigma). DAPI was added to mounting medium. The cells were imaged with a Zeiss Axioplan2 equipped with Q-imaging MP3.3 RTV color camera running QED capture software.

### Electron microscopy

Cells were grown on chambered permanox slides (Electron Microscopy Sciences [EMS], Lansdale, PA), rinsed once with PBS and were immersion fixed overnight with 2% paraformaldehyde and 2% glutaraldehyde in .01 M sodium cacodylate solution (EMS) at 4 degrees C. Sections were rinsed in 0.1 M sodium cacodylate buffer, followed by a quick rinse with ddH2O. Cells were treated with 1% osmium tetroxide/dH20, followed with 2% uranyl acetate/dH20. Cells went through an ethanol dehydration series (25% EtOH/dH20 up to 100% EtOH) and infiltrated through an ascending EtOH/resin series (Embed 812 Kit, EMS), and placed in pure resin overnight. Chambers were separated from the slides, and a modified BEEM embedding capsule (EMS) was placed over defined areas containing cells. Capsules were filled with a drop of pure resin, and placed in vacuum oven to polymerize at 60 degrees C for several hours. Epon resin was added to fill the capsules, and polymerized for 72 h in the vacuum oven. Immediately post polymerization, capsules were snapped from the substrate to dislodge the cells from the slide. Semithin sections (0.5–1 μm) were obtained using a Leica UC7 ultramicrotome (Leica, Buffalo Grove, IL), counterstained with 1% Toluidine Blue, cover slipped and viewed under a light microscope to identify successful dislodging of cells. Ultra-thin sections (80 nms) were collected on copper 300 mesh grids (EMS) using a Coat-Quick adhesive pen (EMS). Sections were counter-stained with 1% uranyl acetate and lead citrate, imaged on an Hitachi 7700 electron microscope (Hitachi High-Technologies,Tokyo, Japan) using an advantage CCD camera (Advanced Microscopy Techniques, Danvers, MA). Images were adjusted for brightness and contrast using Adobe Photoshop CS4 11.0.1.

### Quantitative real-time PCR analysis

Total RNA was prepared from glomeruli and cell lysates using Qiagen RNeasy mini columns (Qiagen, CA) and then reverse transcribed with Superscript-II reverse transcriptase (Invitrogen, CA). cDNA amplification was performed using SYBR-Green PCR Master Mix (Applied Biosystems) and gene-specific exon–exon junction spanning primers (sequences available upon request) in an ABI-Prism 7900HYT Sequence Detection System and evaluated using S.D.S 2.0 software (Applied Biosystems, CA). Normalization to beta-actin gene.

### Lesion frequency - Long QPCR

total DNA was isolated using Qiagen DNeasy mini columns (Qiagen). DNA lesion frequencies were calculated as described [36].

### Oxygen consumption and extracellular acidification rate measurement

cells were plated in collagen I-coated XF24-well microplates (Seahorse Bioscience, North Billerica, MA) in RPMI medium and incubated at 37 °C with 5% CO_2_ for overnight. The oxygen consumption rate (OCR) and extracellular acidification rate (ECAR) were analyzed with the Seahorse Bioscience XF24 Extracellular Flux Analyzer in basal conditions and after injections of 1 μM FCCP (carbonyl cyanide 4-(trifluoromethoxy) phenylhydrazone). Reserve OCR was calculated as the ratio between maximal OCR (FCCP) and average of three baseline OCR measurements [37] to give a reliable and unbiased comparison between highly variable samples.

### Flow cytometry

Fluorescence assay for mitochondrial superoxide: was measured in cells by MitoSOX (Molecular Probes, Carlsbad, CA) as per manufactures instructions.

Viability and Cell death: Two-color flow cytometry was performed as described in [38], using Annexin V-FITC with propidium iodide (BD Pharmingen, San Jose, CA). 10,000 cells were assayed by a BD FACSCanto (BD Biosciences).

*Statistics:* Differences between data groups were evaluated for significance using independent t test or one-way ANOVA. A *p* < 0.05 was considered significant. Correlation analysis was measured by Pearson product-moment correlation coefficient.

## Results

### Mitochondrial oxidative stress and dysfunction of glomerular endothelial cells exposed to diabetic milieu

**Fig. 1:**
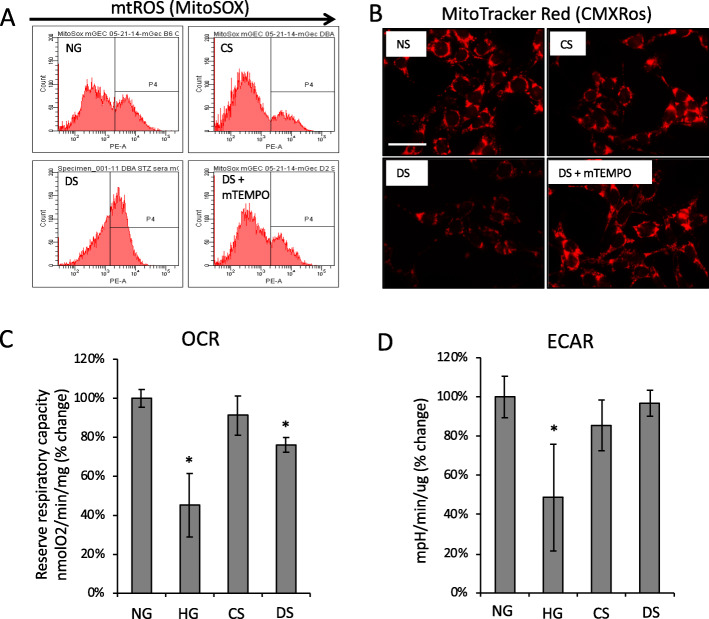
Diabetic milieu induces mitochondrial oxidative stress and decreases mitochondrial function of glomerular endothelial cells. **a** Representative histograms of MitoSOX low and bright fluorescence in mGEC control cells in normal glucose (NG; 5.5 mM glucose) or after culture with either control serum (CS), or diabetic serum (DS) for 24 h without or with mitoTEMPO (mTEMPO; 5μg/ml). **b** Representative images of mitochondrial networks detected by mitoTracker Red in NG, CS, DS treated mGECs without or with mTEMPO. Scale = 50 μm. **c** Mitochondrial respiratory reserve capacity was measured by oxygen consumption rate (OCR; Uncoupled respiration (FCCP) over basal respiration) and glycolysis as determined by measurements of **d** extracellular acidification rate (ECAR) in NG, high glucose (HG; 30 mM glucose), CS, DS treated mGECs. Bars represent mean ± s.e.m. of 3–4 independent experiments; *P < 0.05 versus NG controls

We investigated the effects of diabetic microenvironment or milieu on mitochondrial stress, damage and function of glomerular endothelial cells (GECs). Previous studies show that high glucose (HG) increases mitochondrial superoxide production in murine glomerular endothelial cells (mGECs) [13]. Using MitoSOX assay, we measured mitochondrial superoxide production in mGECs cultured for 24 h with diluted blood serum (2.5% v/v). The serum was collected from non-diabetic DBA2/J mice (control serum: CS), or 6 week diabetic DBA2/J mice (a DKD susceptible strain; diabetic serum: DS) (Fig. 1a), from non-diabetic C57BL/6 mice (a DKD resistant strain) (control serum: CS-B6), or 6 week diabetic C57BL/6 mice (diabetic serum DS-B6) (Supplemental Fig. 1A). Compared to cells grown in regular growth media; normal glucose (NG), CS treatment did not increase the number of MitoSOX positive cells (22 to 19% respectively), however DS treatment markedly increased the number of MitoSOX positive cells to 49% (Fig. 1a). In contrast in either co-incubation with CS-B6 nor DS-B6 increased the number of MitoSOX positive cells (25 and 25.6% respectively, Supplemental Fig. 1A). mGECs cultured in the presence of DS also showed an increase in mitochondrial fragmentation/fission and decreased membrane potential as determined by mitoTracker (Fig. 1b). Co-incubation of DS with mitoTEMPO, a specific scavenger of mitochondrial superoxide production, reduced superoxide and mitochondrial fragmentation (Fig. 1a and b). We measured cellular OXPHOS by quantifying oxygen consumption rate (OCR) and glycolysis (extracellular acidification rate; ECAR) in mGECs. For these experiments mGECs were also treated with normal glucose in the presence of an osmotic control (NG; 5.6 mmol/L glucose + 24.5 mmol/L mannitol), or high glucose (HG; 30 mmol/L glucose). Compared to NG, HG significantly decreased OCR and ECAR significantly after 24 h (Fig. 1c and d). CS had no significant effect on mGEC OCR or ECAR, however, DS significantly reduced OCR in mGEC by 25%, although it did not affect ECAR in mGECs (Fig. 1c and d). OCR and ECAR were not significantly changed in mGECs treated with CS-B6 or DS-B6 compared to NG (Supplemental Fig. 1B, 1C). These results demonstrate that mGECs exposed to serum derived from diabetic DBA2/J mice, susceptible to DKD, can induce mitochondrial superoxide production, mitochondrial fragmentation and disrupted mitochondrial respiration.

### Diabetic milieu caused accumulation of mtDNA lesions, of endonuclease G in the inner mitochondrial membrane space (IMS), and disrupted mitochondrial architecture in glomerular endothelial cells

**Fig. 2:**
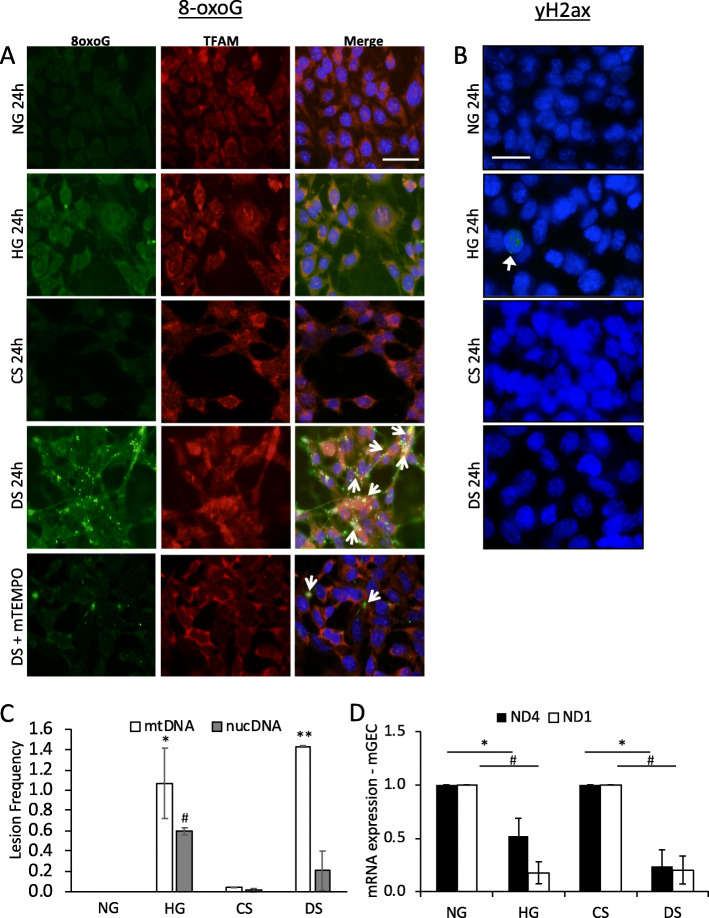
Diabetic milieu induced increased mtDNA oxidative lesions but not nuclear (nuc) DNA lesions. **a** Representative immunofluorescence images detecting DNA 8-oxoG (green) and mitochondrial transcription factor A (mTFA; red) and DAPI in mGECs treated with NG, HG, CS, DS without or with mTEMPO for 24 h. Open arrows in A depict clusters and colocalization of 8-oxoG and TFAM. Arrows in B show mGEC nucleus with positive γ-H2AX foci. Scale = 100 μm. **b** Representative immunofluorescence images detecting γ-H2AX foci (green) and nuclei (DAPI blue) in mGECs treated with NG, HG, CS and DS for 24 h. Scale = 100 μm. Quantification of lesion frequencies in mtDNA and in nuclear (nuc) DNA by QPCR assay in mGECs treated 24 h with NG, HG, CS and DS (*n* = 3 ± s.e.m. relative amplification normalized to non-damaged NG controls; * *P* < 0.05 or ** *P* < 0.01 versus mtDNA control, #*P* < 0.05 versus nucDNA control). **d** RT-PCR quantification of transcripts for mitochondrial encoded genes; ND1 and ND4 in mGECs cultured in NG, HG, CS and DS for 24 h. Bars represent mean ± s.e.m. of 4 independent experiments; ND4 *P < 0.05 versus NG or CS controls, ND1 #*P* < 0.05 versus NG or CS controls

We next examined oxidative stress damage by assessing DNA 8-Oxoguanine (8-oxoG), a common DNA modification resulting from ROS damage [39]. Figure 2a shows that HG specifically increased the presence of cytoplasmic mtDNA 8-oxoG positive staining, co-localized to mitochondrial transcription factor A (TFAM1) a marker for mtDNA. Furthermore, an increase in mtDNA 8-oxoG co-localized to TFAM1 was also detected in mGEC cultured in DS but not in CS. Interestingly, 8-oxoG in presence of DS was observed as puncta and clusters throughout the cytoplasm (Fig. 2a- arrows). Co-incubation with mitoTEMPO reduced mtDNA 8-oxoG observed in DS treated mGECs (Fig. 1c, bottom panels). mGECs treated with serum from DKD resistant C57BL/6 mice; CS-B6 or DS-B6 did not show positive staining for 8-oxoG (Supplemental Fig. 1D).

Evidence for DNA double strand breaks (DSBs) in nuclear DNA have been reported in patients with long term diabetes [40]. We assessed nuclear DSBs in mGECs by detecting the histone H2A variant; γ-H2AX, which serves as a sensitive indicator of nuclear DNA DSB formation [41, 42]. HG treatment induced some DSBs in ~ 3–5% of cultured mGECs after 24 h, as detected by the presence of nuclear positive γ-H2AX foci (Fig. 2b). There were no positive γ-H2AX foci detected in mGECs treated with CS or DS after 24 h. With sustained HG treatment (48 h and 72 h), the majority of mGECs showed nuclear γ-H2AX foci (Supplementary Fig. 2A, B respectively). To validate our responses in mGECs, we performed a short pulse (10 min) of H_2_O_2_ (10 μM) known to only induce mtDNA oxidative damage in cancer cell lines [43], as a positive control (Supplementary Fig. 2C and 2D). Positive cytoplasmic 8-oxoG staining but no γ-H2AX positive foci in mGECs were detected. However, with longer H_2_O_2_ treatment (60–90 min), we observed increased number of cells with γ-H2AX positive foci as well as nuclear condensation (Supplementary Fig. 2C and 2D). We used a QPCR assay to measure DNA lesion frequency in nuclear (nuc) DNA and in mtDNA [36] of mGECs. HG increased significant mtDNA and nucDNA lesions after 24 h of treatment (Fig. 2c). There were no lesions detected in CS treated mGECs compared to NG. However, there were significantly more mtDNA lesions detected after 24 h of DS treatment compared to nuclear lesions or all lesions in CS treated cells (Fig. 2c). Increased mtDNA damage was associated with a significant decreased in the transcription of mitochondria encoded ND1 and ND4 genes by HG and DS compared to controls (Fig. 2d). Consistent with 8-oxoG and γ-H2AX staining, a 10 min pulse of H_2_O_2_ resulted in increased mtDNA lesions in mGECs, while nuclear DNA lesions were only detected at longer exposure times (Supplementary Fig. 2E). These positive control data confirm that mtDNA is a target of increased superoxide production by the diabetic milieu affecting mtDNA stability and transcription of essential mitochondrial encoded genes.

**Fig. 3:**
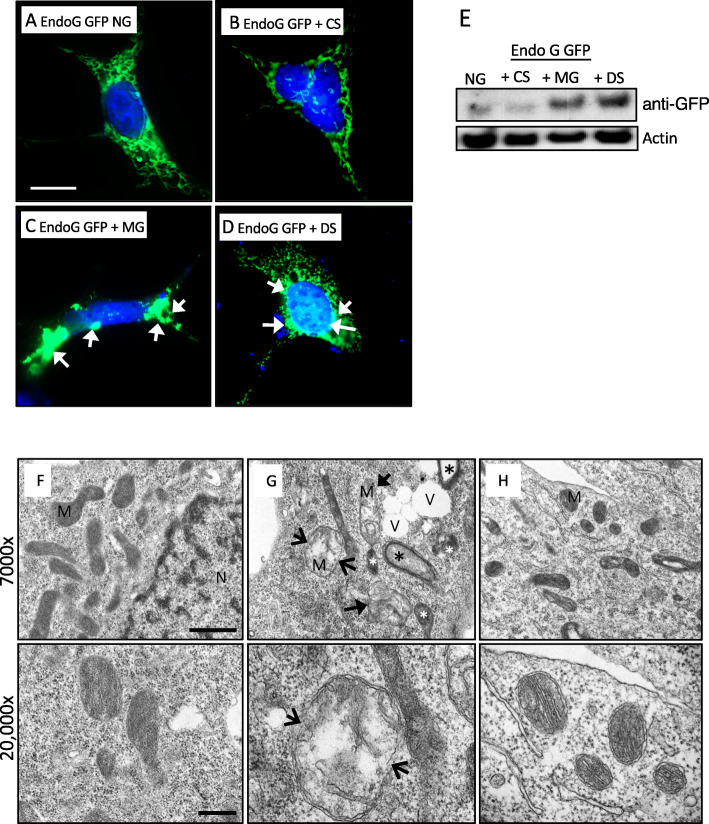
Diabetic milieu induces increased endonuclease G accumulation in mitochondrial intermembrane space (IMS) and disrupts mitochondrial architecture. **a** Immunofluorescence detection of endoG-GFP located in the IMS of mGECs cultured in NG, **b** with CS, **c** MG132 and with **d** DS for 48 h. Arrows show endoG clustering. Scale = 20 μm. **e** Western blot detecting anti-GFP from endoG-GFP whole cell extracts in **a**-**d** with β-actin shown as a loading control (*n* = 3). **f** Representative transmission electron microscopy images (magnification 7000x, Scale = 1 μm) showing mitochondria (M) in mGEC cultured in NG control shown as elongated mitochondria with dense matrix and densely aligned cristae, N represents nucleus. Below is a higher power images (12,000x, Scale = 0.5 μm), showing clear mitochondrial inner and outer membranes. **g** Mitochondrial matrix in DS treated mGECs is largely reduced and few cristae remain, outer membrane rupture is seen (open arrow). There are triple and quadruple membrane rings indicative of autophagy and/or mitophagy (*), and a large number of vacuoles were evident (V) and mitochondria with electron dense material (white *). H) DS + mTEMPO treated mGECs show improved mitochondrial architecture with intact outer membrane and inner membrane cristae

The increased mitochondrial superoxide in mGECs exposed to DS could also be detrimental to the mitochondrial intermembrane space (IMS), where oxidation can lead to protein misfolding and aggregation [44–46]. Endonuclease G (endoG) is an IMS protein important in the initiation of apoptosis and has been described to accumulate in clusters and to promote mitochondria condensation in the IMS upon mutation or inhibition of the proteasome [47]. We therefore examined endoG by transfecting mGECs with endoG-GFP construct [47] and examined its distribution in the cells by fluorescent microscopy. Figure 3a shows mGECs with endoG in NG had the expected mitochondria distribution throughout the cytosol as networks. Similar distribution was observed in CS treated cells (Fig. 3b). When inhibiting the 26S proteasome with MG132 (1 μM, 20 h; Fig. 3c), there was prominent clustering and perinuclear distribution of endoG in the cells. mGECs in the presence of DS showed fragmentation of networks, cytoplasmic clustering and perinuclear distribution of endoG (Fig. 3d). EndoG-GFP in cell extracts was confirmed by western blotting showing an increase in MG132 and DS treated mGECs compared to NG or CS (Fig. 3e) suggesting abnormal accumulation and that DS can impact mitochondrial proteostasis of mGECs.

Next, we examined the mitochondrial morphology of mGECs by transmission electron microscopy. Compared to controls, DS treatment resulted in substantial disruption of inner mitochondrial membranes, loss of cristae, changes in mitochondrial matrix density with areas of electron-dense material (Fig. 3g; white *), and outer membrane rupture (Fig. 3g; open arrows). There was evidence of increased number of vacuoles as well as triple and quadruple membrane rings consistent with the presence of autophagy and/or mitophagy (Fig. 3g, V and black * respectively). The DS-associated damage was largely ameliorated by stabilization of mitochondrial superoxide with mitoTEMPO treatment (Fig. 3h). In this setting, mitochondria had more visibly regular structures of cristae and intact double outer mitochondrial membranes.

These results suggest that the diabetic milieu impacts mitochondria of glomerular endothelial cells directly, causing DNA damage exclusively in mtDNA, decreased transcription of essential mitochondrial genes, promoting the aggregation of IMS proteins and disrupting mitochondrial architecture. MitoTEMPO prevented these changes suggesting that increased mitochondrial superoxide in diabetes contributes to this phenotype. We next explored whether the increased mitochondrial superoxide damage by the diabetic microenvironment had functional implications for endothelial cells.

### Diabetic milieu causes glomerular endothelial cell dysfunction but not cell death

**Fig. 4:**
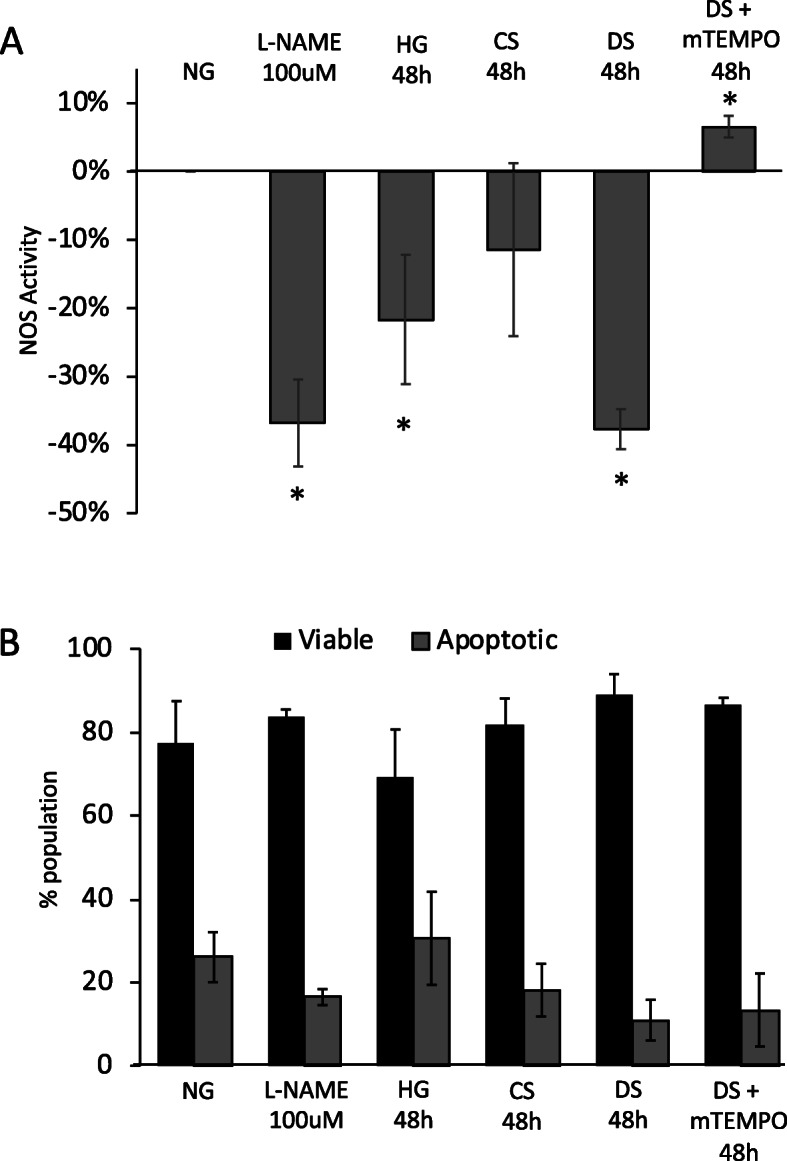
Diabetic milieu induces endothelial cell dysfunction without cell death. **a** Nitric oxide synthase (NOS) activity detected by DAF-FM fluorescence in mGEC cultured in NG control conditions without or with L-NAME (100 μM), or cultured with HG, CS, DS and DS + mTEMPO for 48 h. **b** Percentage viable (Annexin and PI negative) and apoptotic (Annexin and PI positive) mGECs cultured in the same conditions as **a** for 48 h. Bar graphs represent *n* = 4–5 ± s.e.m. averaged independent experiments; *P < 0.05 versus NG controls

Mitochondrial dysfunction in endothelial cells causes endothelial cell dysfunction, characterized by decreased bioavailability of nitric oxide (NO) and inhibition of nitric oxide synthase (NOS) [48]. In our studies, NOS activity in mGECs was significantly decreased by NOS inhibitor L-NAME, HG and DS treatment (Fig. 4a). Co-incubation of DS with MitoTEMPO prevented the decrease in NOS activity, and the levels in this group were greater than those in NG controls. A decrease in NOS activity by L-NAME, HG and DS treatments did not coincide with changes in cell viability after 48 h (Fig. 4b). These results demonstrate that diabetic milieu induces mitochondrial oxidative stress and dysfunction, which is sufficient to induce endothelial dysfunction of mGECs but not cell death.

### Diabetic milieu caused autophagy flux in glomerular endothelial cells

**Fig. 5:**
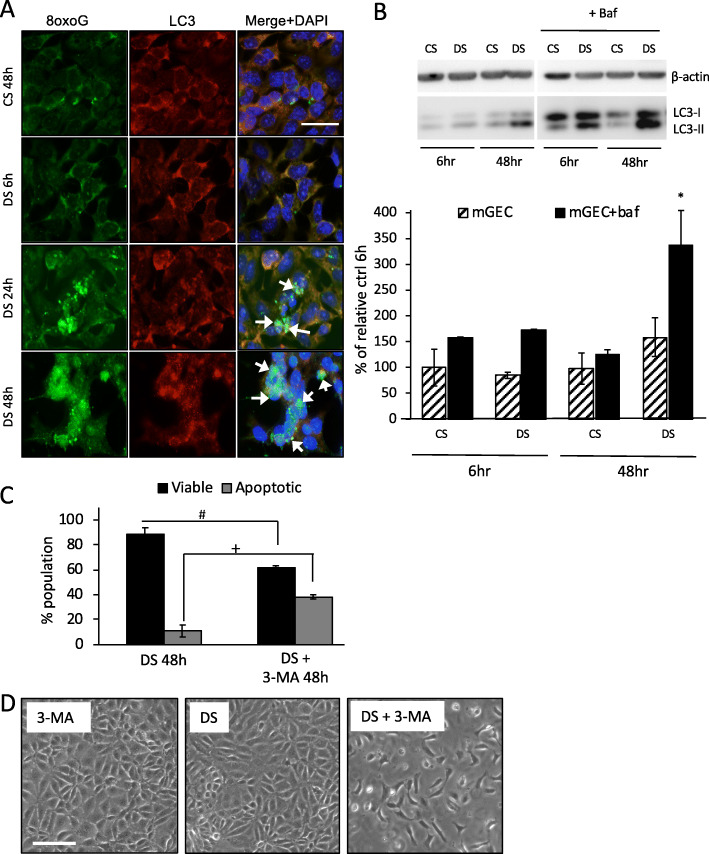
Diabetic milieu induces autophagy flux in mGECs. **a** Representative immunofluorescence images detecting 8-oxoG (green), LC3 (red) and nucleai with DAPI (blue) in mGEC cultured with CS, or cultured with DS for 6, 24 and 48 h. Arrows depict clustering and colocalization of 8-oxoG and LC3 puncta. Scale = 100 μm. **b** western blot show the ratio of LC3-II/LC3-I protein levels in lysates from mGECs cultured in CS or DS for 6 and 48 h in absence (black and white bars) or presence of 6 h bafilomycin A1 (Baf, 100 nM; black bars). **c** Percentage viable (Annexin and PI negative) and apoptotic (Annexin and PI positive) mGECs cultured in DS without or with 3-MA for 48 h. **d** Representative contrast images of mGECs treated with 3-MA, or DS without or with 3-MA for 48 h. Bar graphs represents *n* = 4 ± s.e.m. independent experiments; *P < 0.05 versus DS-Baf in **b**, viability #*P* < 0.05 versus DS and apoptotic +*P* < 0.05 versus DS

We investigated the molecular basis of mitochondrial turnover in stressed mGECs. For these studies mGECs were treated with CS and DS to examine autophagy after chronic exposure (48 h). The microtubule associated protein 1 light chain 3 (LC3), is a central protein for autophagy involved in substrate selection and autophagosome biogenesis [49]. mGECs exposed to DS had increased mtDNA 8-oxoG staining at 6 h, and 8-oxoG lesions increased and accumulated after 24 and 48 h (Fig. 5a). There was a concomitant increase in LC3 consistent with an increase in autophagy (Fig. 5a-arrow show colocalization). We next tested LC3 turnover to determine flux, based on the observation that LC3-II is degraded in autolysosomes [49]. mGECs were treated with or without bafilomycin A1 (baf; 100 nM) for 6 h after 42 h with 2.5% v/v of either DS or CS for total 48 h and calculated the ratio of LC3-II/LC3-I protein levels by western blotting Fig. 5b). Baf inhibits the acidification inside the lysosome or the autophagosome-lysosome fusion. Baf treatment resulted in the accumulation of LC3-II in cells treated with CS (Fig. 5b). At 48 h, DS treatment increased LC3-II, and greater LC3-II levels were observed in DS + Baf (Fig. 5b). These results suggest that there is increased autophagic flux in mGECs treated with DS. This is consistent with observations from TEM sections in Fig. 3g. Furthermore, inhibition of autophagy in mGECs with 3-Methyladenine (3-MA; type III phosphatidylinositol 3-kinases inhibitor; 5 mM), resulted in a significant increase in cell death compared to DS treatment (Fig. 5c,d). These results suggest increased autophagic flux is a possible stress adaptive response.

### Glomerular endothelial cell stress by diabetic milieu resulted in secretion of pro-apoptotic factors into supernatants affecting podocytes

**Fig. 6:**
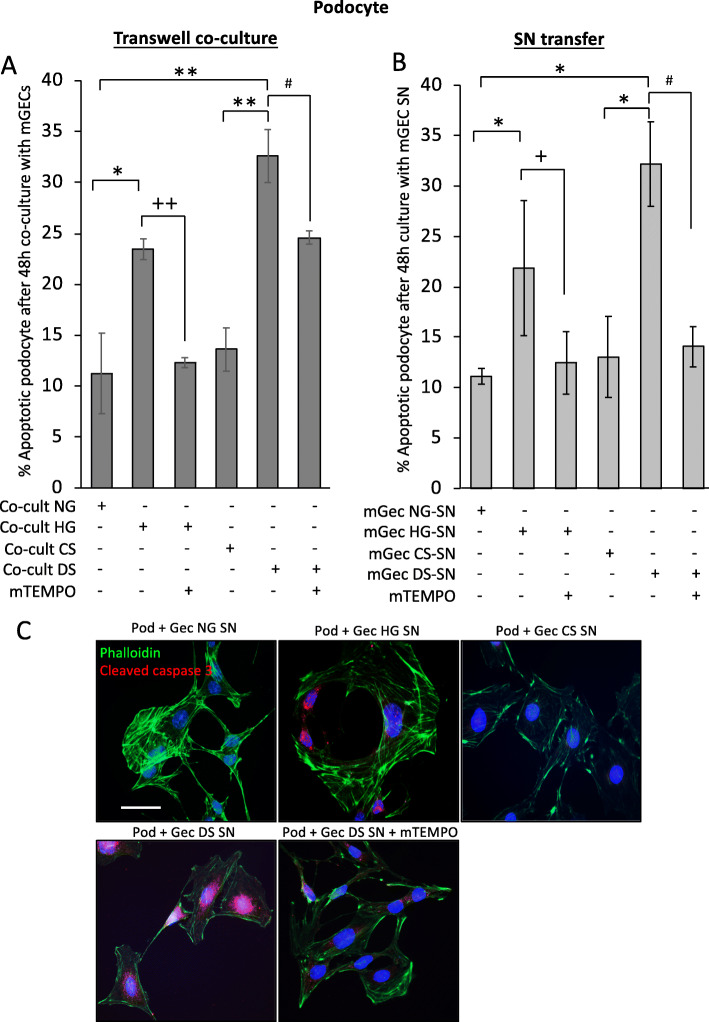
mGEC exposed to diabetic serum secrete factors that cause podocyte cell death. **a** Percentage podocyte cell death (AnnexinV-FITC and PI positive cells) 24 h after co-culture with mGEC in transwell inserts; without or with HG, or HG in the presence of mitoTEMPO (5μg/ml), or CS, or DS, or DS + mTEMPO for 24 h (*n* = 4). **b** Percentage apoptotic podocytes after supernatant transfer (SN) from mGECs; NG-SN, or HG-SN, or HG + mTEMPO-SN, or CS-SN, or DS-SN, or DS + mTEMPO-SN. MitoTEMPO dose used was 5 μg/ml (*n* = 3). **c** Representative immunofluorescence images detecting cleaved caspase 3 (red), and F-Actin (phalloidin; green) and nuclei (DAPI; blue) in podocytes cultured with NG-SN, or HG-SN, or CS-SN, or DS-SN, or DS + mTEMPO-SN for 24 h. Scale = 50 μm. Cultures were performed in 10% FCS. Bars in **a** and **b** represent averaged independent experiments ± s.e.m.; *P < 0.05, **P < 0.01 versus NG or CS controls, +*P* < 0.05, ++*P* < 0.01 versus HG, #*P* < 0.05 versus DS

Glomerular endothelial cell injury in early DKD plays an important role in its pathogenesis [50]. We have demonstrated that oxidative stress and damage accumulation in mitochondria of GECs is key in DKD susceptibility in mice [13]. In order to examine crosstalk of stressed mGEC and podocytes, we used a Transwell co-culture system with podocytes and mGECs. We examined podocyte cell death by FACS (AnnexinV/PI) and in adjacent mGEC we measured mitochondrial superoxide (MitoSOX) after 24 h co-culture. Podocyte apoptosis was significantly increased in co-cultures with mGECs in the presence of HG compared to NG control co-culture, and apoptosis was significantly prevented in the presence of mTEMPO (Fig. 6a). Podocyte apoptosis was significantly increased in co-cultures with mGECs in the presence of DS compared to CS, and apoptosis was significantly prevented in the presence of mTEMPO (Fig. 6a). The mean fluorescence intensity (MFI) for mitochondrial superoxide in mGECs in Tranwell inserts increased significantly from 1730.3(±179) in NG co-cultures to 2301.5(±250, *P* < 0.01) in HG co-cultures. MFI of superoxide was reduced to 1738.5(±148, *P* < 0.05) in HG + mTEMPO. mGECs in Transwells had significantly increased mitochondrial superoxide when cocultured in the presence of DS with MFI of 2744.3 ± 455, from 1923.3 ± 116 in CS (*P* < 0.01), and superoxide was significantly reduced to 2327(±189, *P* < 0.05) in DS + mTEMPO. In the Transwell setting there is a by-directional influence from both cell types in the co-cultures, therefore in order to determine the effect of stressed mGECs on podocytes, we next examined cell death in podocytes incubated with the supernatant (SN) derived from mGECs treated with normal glucose (NG-SN), high glucose (HG-SN), without or with mitoTEMPO, or SN from control serum (CS-SN), and diabetic serum (DS-SN), without or with mitoTEMPO. The percentage of podocyte death was increased significantly in cultures with HG-SN compared to NG-SN controls (Fig. 6b, Supplemental Fig. 4A). Under control conditions with NG-SN, podocytes displayed typical morphology features such as large, flat arborized cellular shape with well-developed neurite-like extensions and microvilli, however HG-SN treatment caused cell shrinkage, resulting in an aster-like cell shape (Supplemental Fig. 4B). Interestingly, supernatant derived from mGECs treated with HG and mitoTEMPO did not induce podocyte death (Fig. 6b, Supplemental Fig. 4B). In contrast to CS-SN treated podocytes, DS-SN mediated a profound increase in podocyte death (32%; Fig. 6b, Supplemental Fig. 4A) and in culture, podocytes were characterized by cell shrinkage and some detachment (Supplemental Fig. 4B). This paracrine effect was prevented in supernatants from mGECs treated with DS and mitoTEMPO, and the levels were comparable to NG-SN and CS-SN controls (Fig. 6b, Supplemental Fig. 4B). Staining for cleaved caspase 3 confirmed cell death by apoptosis of podocytes in HG-SN and DS-SN co-cultures (Fig. 6c). Disruption of the phalloidin-labeled filamentous actin (F-actin) network was also consistent with podocyte injury mediated by HG-SN and DS-SN (Fig. 6c). No caspase 3 staining was detected in podocytes co-cultured with DS-SN + mTEMPO (Fig. 6c).

Although podocytes do not interact directly with blood components in vivo, we determined the direct effects of HG, CS and DS on podocytes in complete media (10% FBS). Neither treatment induced significant cell death by AnnexinV/PI staining (Supplemental Fig. 4A). Immunoblot analysis of cells lysates showed no evidence of cleaved caspase-3 in NG, HG, CS or DS treated podocytes, in contrast to podocytes grown in 0.2% FCS treated for 24 h with 5 ng/ml of TGF-β showing cleavage of caspase-3 (positive control; Supplemental Fig. 4B). Together the in vitro results from both direct co-culture and media transfer studies, demonstrate consistently and at a molecular level that diabetic microenvironment causes endothelial dysfunction of mGEC, which was necessary and sufficient to increase podocyte apoptosis. These data support the emerging notion that a molecular signaling crosstalk between stressed mGECs and podocytes exists, and point to the importance of mitochondria stress in mGECs as key mechanism enabling the release of secreted factors that can affect podocytes directly.

## Discussion

Understanding the mechanisms by which diabetes affects glomerular endothelial cells and their redox state and the impact on neighboring glomerular cells can help define novel cell specific targets for preventing glomerular injury in DKD. In this study we examined glomerular endothelial cell stress and dysfunction after exposure to high glucose (HG) and diluted factors in the circulating blood of non-diabetic control (CS) and 6 week diabetic (DS) DBA2/J mice that are susceptible to developing DKD. Here we show that treatment of mGECs with HG or DS resulted in increased mtROS, oxidative mtDNA damage, affecting mitochondrial proteostasis evident by accumulation and aggregation of proteins in the IMS, induced mitochondria fission, loss of cristae, membrane rupture and reduced mitochondrial encoded gene expression and overall mitochondrial function compared to corresponding controls and these effects could be ameliorated by a mitochondrial superoxide specific scavenger (mitoTEMPO). Hence the diabetic environment can mediate GEC dysfunction resulting in decreased NOS activity by triggering mitochondria oxidative stress. Treatment of mGECs with DS from mice that are resistant to developing DKD (C57BL/6), did not exert oxidative stress or affect mitochondrial function, suggesting that composition of circulating factors in the diabetic milieu differs between strains with differing susceptibility to development of DKD. Onset of disease in predisposed individuals is key to drive mitochondrial dysfunction and DKD progression as previously demonstrated in studies of cells exposed to sera from patients collected prior to the development of DKD and with confirmed DKD [51, 52]. In mice we have previously reported that oxidative damage in mtDNA accumulates over time specifically in GECs of DKD susceptible mice [13]. Our new results suggest that chronic exposure of GECs to the diabetic milieu from DKD susceptible mice results in excessive mitochondrial derived superoxide production, and a steady state accumulation of oxidized products that cause cellular dysfunction, which in turn promote glomerular injury.

Soluble factors in the conditioned supernatants from stressed mGECs by either treatment with HG or DS promote podocyte apoptosis by Tranwell co-cultures or supernatant transfer experiments. This is important because HG or DS do not directly cause podocyte apoptosis in complete media, supporting the notion that proapoptotic paracrine factors are being secreted by stressed GECs. By scavenging mitochondrial superoxide in DS treated mGECs, we were able to prevent podocyte apoptosis in culture. The nature of the paracrine signals in the SN that mediate podocyte cell death are the subject of current investigation by our laboratory. Nevertheless, studies investigating secreted exosomes derived from high glucose treated GECs have demonstrated their ability to mediate EMT and podocyte dysfunction in a paracrine manner via canonical Wnt/β-catenin signaling [53], and to promote α-smooth muscle actin (α-SMA) expression, proliferation and extracellular matrix protein overproduction in mesangial cells through the TGF-β1/Smad3 signaling pathway in vitro [54] and in vivo [55]. These reports are in agreement with our observations, that GEC responses to high glucose as well as other factors in the diabetic milieu result in the secretion of pro-apoptotic factors potentially driving a pathogenic intercellular crosstalk in the glomerulus.

Upstream from the secretion of these factors is mitochondrial stress and endothelial dysfunction triggered by the diabetic milieu. While energy depletion may be an adequate explanation for mitochondrial-related injuries, considering the coexistence of the three glomerular cell types in vivo, in particular from the point of view of vasculature, it is possible that mitochondrial function in GECs has a regulatory nature. Other than energy production, mitochondria are important regulator of redox balance and calcium homeostasis and these metabolic tasks are intertwined with their role as modulator of regulatory pathways such as cell death, biosynthesis or metabolism of lipids, amino acids and iron sulfur (Fe-S) clusters, and immune responses [56]. Unexpectedly, despite the highly oxidative environment, there was an adaptive response allowing cells to survive after chronic exposures with DS (even after 72 h; not shown) and consequently resulting in GEC dysfunction by reduced NOS activity and therefore reduced NO production. It is known that mitochondrial stress can stimulate the synthesis of specific mitochondrial proteins by activating mitochondrial biogenesis to balance the impact of mitochondrial stress on cellular homeostasis [57–59]. In mGECs exposed to DS we detected decreased respiration, an accumulation of oxidized mtDNA lesions as well as aggregation of endonuclease G possibly through protein misfolding in the IMS. We provide evidence that this inadequate accumulation of posttranslational modifications arising from an oxidative stress has implications in cellular function, and may be facilitated by increased autophagy, which if impaired through chemical blockade of autophagic-lysosomal degradation, resulted in increased cell death of the stressed GECs. Importantly, alteration of autophagy by endothelial cell specific knock out of Atg5 in diabetic mice resulted in capillary rarefaction, glomerular basement membrane thickening, podocyte foot process effacement and accelerated DKD [60]. With this knowledge combined with reports demonstrating that well-known inducers of autophagy are protective, although through not exclusive pathways, and have been shown to have positive effects on inflammation, tubular injury, glomerulosclerosis, and podocyte injury in experimental DKD [61–63], hence potentiating this pathway has been beneficial for preventing kidney injury [64, 65]. However the survival of these dysfunctional glomerular endothelial cells can consequently activate mechanisms leading to the secretion of pathogenic soluble factors in DS-SN. By preventing mitochondrial superoxide generation in DS treated mGECs, we were able to prevent the secretion of pathologic crosstalk factors in DS-SN and restored podocyte survival. This is the first step to define and recapitulate key inter-cellular interactions and paracrine communication among GECs and podocytes in a controlled microenvironment, further characterization of the key crosstalk factors, cellular biomechanics and pathophysiology in a physiologically relevant context will help identify potential cell-type-specific targets for therapeutic intervention.

## Conclusion

The integrity of the glomerular filtration barrier is maintained by an interplay between the intercapillary endothelium, the basement membrane and epithelial podocytes. In diabetes crosstalk signaling from the endothelium to podocytes can cause a pathologic signaling cascade contributing to podocyte depletion, albuminuria and glomerulosclerosis in DKD. Identification of the mechanism(s) governing this intercellular communication in the glomerulus will lead to a better understanding of the pathogenesis of DKD and, potentially, to the identification of new therapeutic targets.

## Supplementary information

**Additional file 1.**

## Data Availability

I can confirm I have included a statement regarding data and material availability in the declaration section of my manuscript.

## Abbreviations

8-oxoG: 8-Oxoguanine
CKD: chronic kidney disease
CS: control serum; serum from non-diabetic mouse
DS: diabetic serum; serum from 6 week diabetic DBA2/J mouse
DKD: diabetic kidney disease
FACS: fluorescence-activated cell sorter
FSGS: focal segmental glomerulosclerosis
GBM: glomerular basement membrane
GEC: glomerular endothelial cell
HG: high glucose (30 mM)
mGEC: murine glomerular endothelial cell
MFI: mean fluorescence intensity
mTempo: mitoTEMPO
NG: normal glucose (5.5 mM)
RT-PCR: real time polymerase chain reaction
ROS: reactive oxygen species
SN: supernatant from mGECs
TGF-β: Transforming growth factor-β (TGF-β)
