# Supplementary figures and images for: The diabetic microenvironment causes mitochondrial oxidative stress in glomerular endothelial cells and pathological crosstalk with podocytes
Source: Cell Commun Signal. 2020 Jul 8;18:105. doi: 10.1186/s12964-020-00605-x (PMC7341607; doi:10.1186/s12964-020-00605-x)

Supplementary FIGURE 1

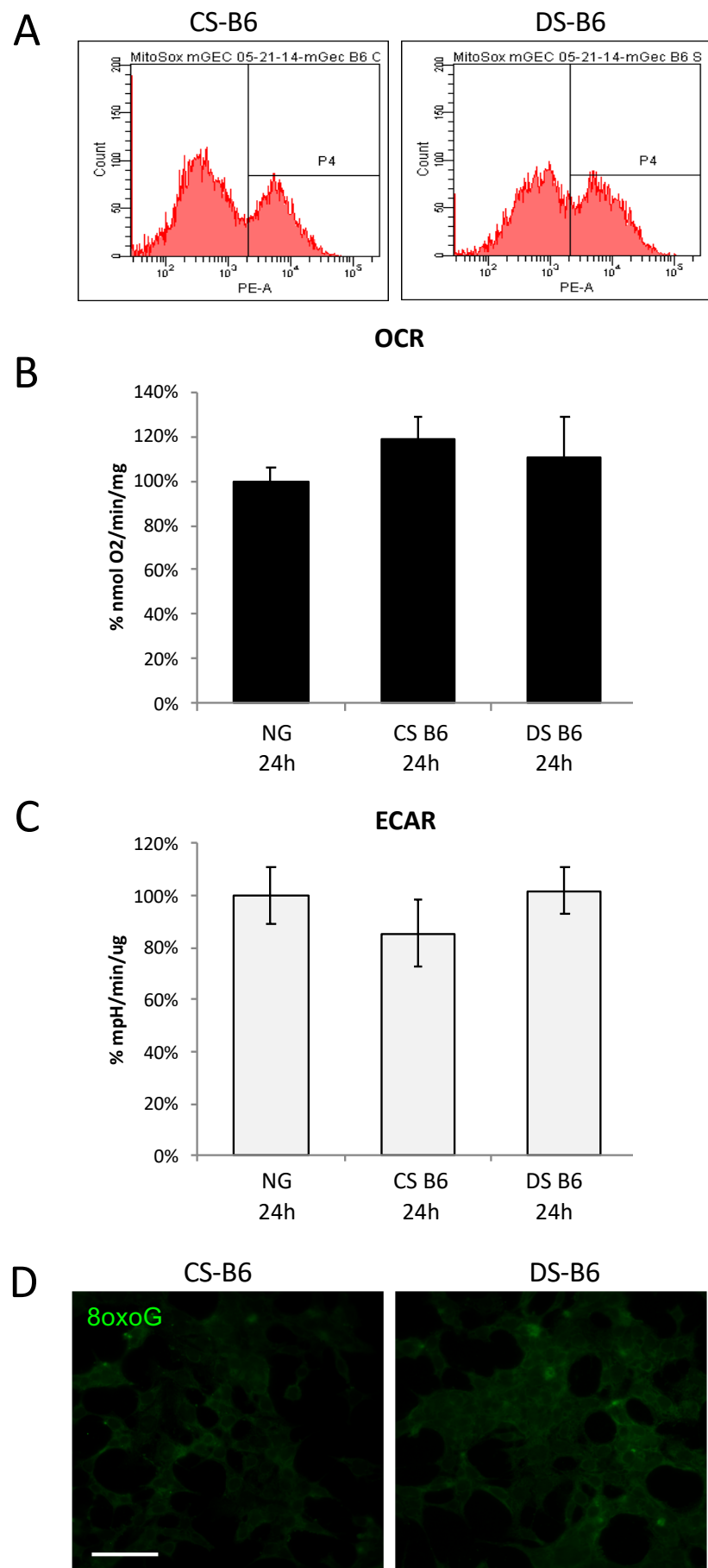

Supplementary FIGURE 2

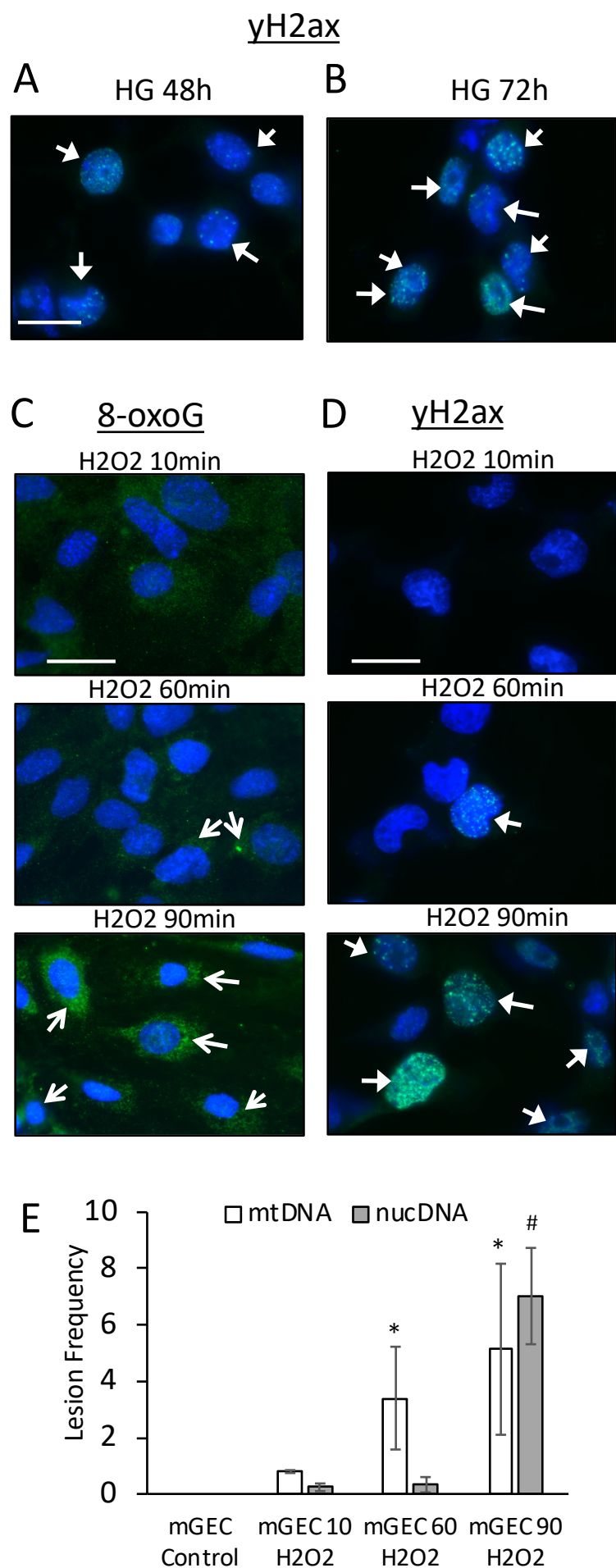

Supplementary FIGURE 3

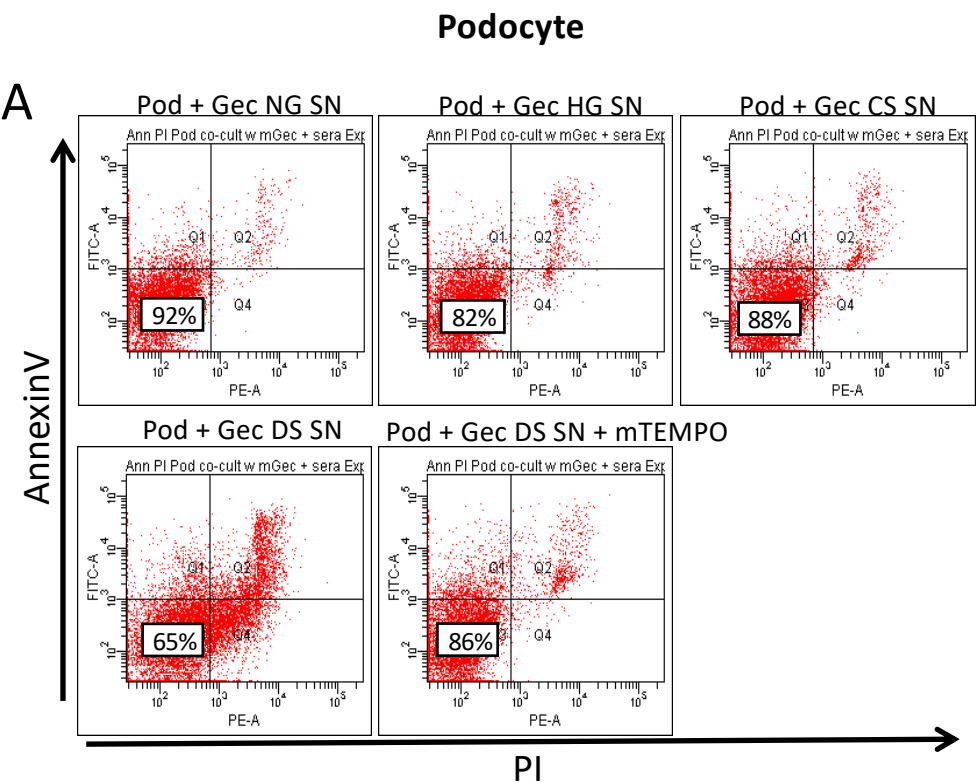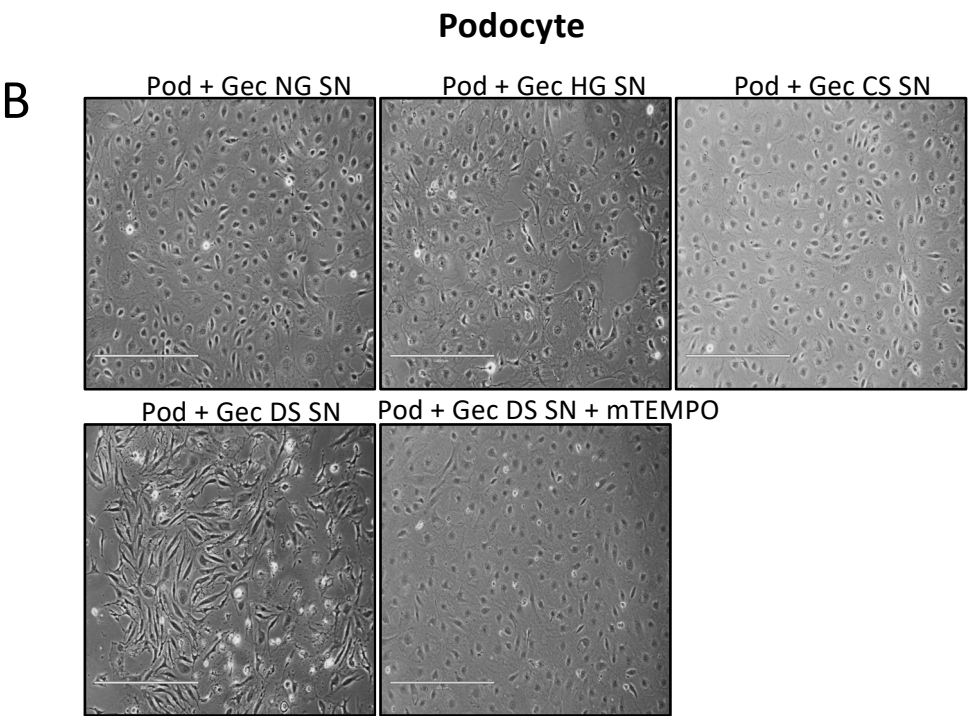

Supplementary FIGURE 4

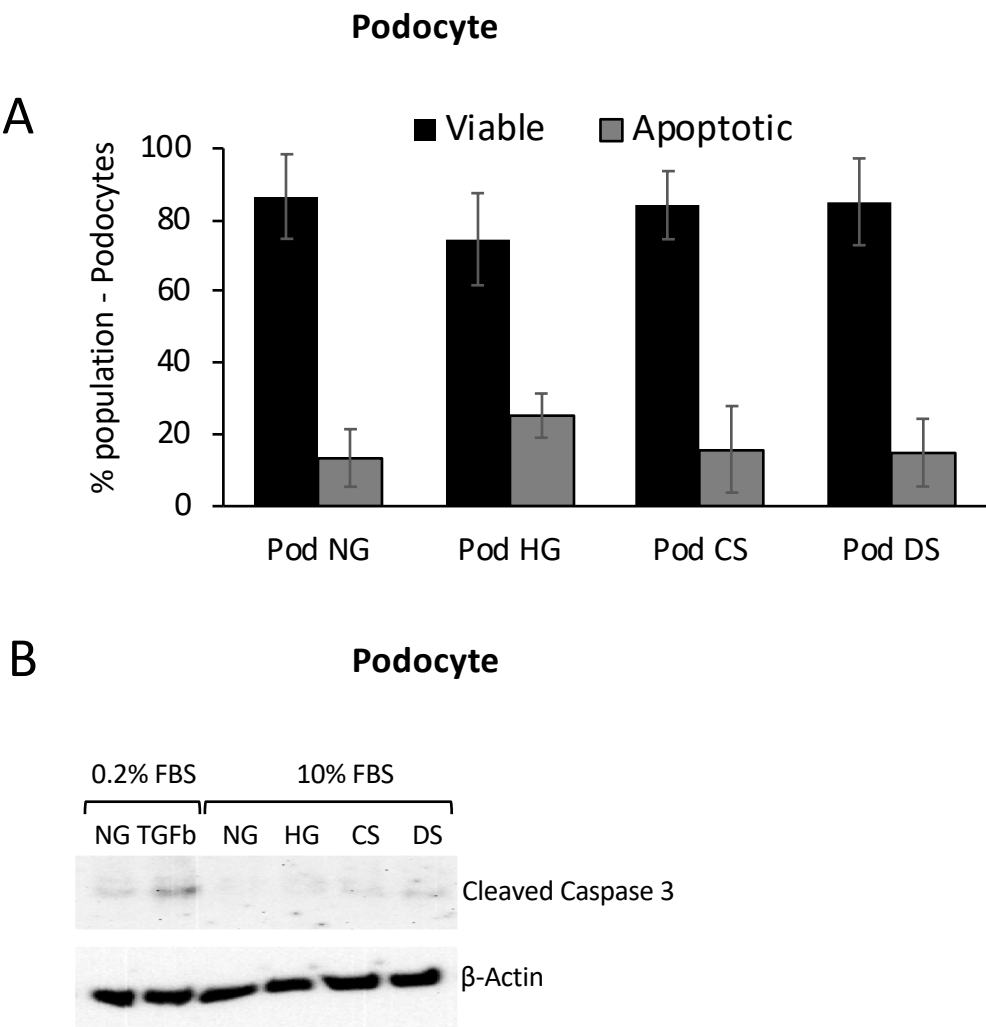

Supplement: Supplementary file 2 — Additional file 1. [file 12964_2020_605_MOESM2_ESM.pdf]
